# Negative pressure wound therapy for soft tissue injuries around the foot and ankle

**DOI:** 10.1186/1749-799X-4-14

**Published:** 2009-05-09

**Authors:** Hyun-Joo Lee, Joon-Woo Kim, Chang-Wug Oh, Woo-Kie Min, Oog-Jin Shon, Jong-Keon Oh, Byung-Chul Park, Joo-Chul Ihn

**Affiliations:** 1 Department of Orthopedic Surgery, Kyungpook National University Hospital, Daegu, Korea; 2 Department of Orthopedic Surgery, Yeungnam University Hospital, Daegu, Korea; 3 Department of Orthopedic Surgery, Korea University Guro Hospital, Seoul, Korea

## Abstract

**Background:**

This study was performed to evaluate the results of negative pressure wound therapy (NPWT) in patients with open wounds in the foot and ankle region.

**Materials and methods:**

Using a NPWT device, 16 patients were prospectively treated for soft tissue injuries around the foot and ankle. Mean patient age was 32.8 years (range, 3–67 years). All patients had suffered an acute trauma, due to a traffic accident, a fall, or a crush injury, and all had wounds with underlying tendon or bone exposure. Necrotic tissues were debrided before applying NPWT. Dressings were changed every 3 or 4 days and treatment was continued for 18.4 days on average (range, 11–29 days).

**Results:**

Exposed tendons and bone were successfully covered with healthy granulation tissue in all cases except one. The sizes of soft tissue defects reduced from 56.4 cm^2 ^to 42.9 cm^2 ^after NPWT (mean decrease of 24%). In 15 of the 16 cases, coverage with granulation tissue was achieved and followed by a skin graft. A free flap was needed to cover exposed bone and tendon in one case. No major complication occurred that was directly attributable to treatment. In terms of minor complications, two patients suffered scar contracture of grafted skin.

**Conclusion:**

NPWT was found to facilitate the rapid formation of healthy granulation tissue on open wounds in the foot and ankle region, and thus, to shorten healing time and minimize secondary soft tissue defect coverage procedures.

## Introduction

Tendon and/or bone exposure commonly occurs in the foot and ankle region after acute trauma [1]. The conventional treatment method used for these uncovered, open wounds in the foot and ankle is skin grafting after the formation of healthy granulation tissue by wet dressing [2]. However, the duration of treatment may be prolonged, and patients may experience severe pain during dressing changes [3]. Furthermore, it is difficult to form healthy granulation tissue by simple wet dressing, when a tendon, bone, or implant is exposed. Accordingly, free flap surgery is often required, which requires substantial effort and introduces the issue of donor site morbidity [4].

Negative pressure wound therapy (NPWT) was first described by Argenta and Morykwas [2]. This technique can be used to cover exposed bone or soft tissue defects without frequent dressing changes, and reduces chronic edema and increases local blood supply, which enhances the formation of healthy granulation tissue. Several reports have been issued on the application of NPWT to soft tissue defects of the extremities, abdomen and chest [5,6]. However, reports regarding its use in the foot and ankle region are limited, though in this region tendon and bone exposures frequently occur after external injury or due to chronic ulcerative disease. The purpose of this study was to determine how NPWT helps healing and whether the technique can reduce the need for flap surgery for the treatment of acute or chronic open wounds in the foot and ankle region.

## Materials and methods

Over the four year period from 2003 to 2006, 16 patients (12 males and 4 females) with soft tissue injuries in the foot and ankle region were treated with an NPWT device (V.A.C.,^® ^Vacuum Assisted Closure, KCI, San Antonio, United States) at the authors' institute. All 16 patients were followed for more than 12 months (mean: 19 months, range: 13–39 months). Mean patient age was 32.8 (range: 3–67). All patients had experienced an acute injury, caused by either a traffic accident in 12, a falling from a height in 2, and a crush injury in 2. Wound locations were on the medial side of the ankle in 3 cases, the lateral side of the ankle in 1 case, and of the dorsum of the foot in 12 cases. All patients had at least one tendon or bone exposed at the initiation of NPWT, and four had an associated infection (Table 1).

**Table 1:** Patient and wound details before and after negative pressure wound therapy

**No**	**Age**	**Sex**	**Injury**	**Site**	**Wound grade(before)**	**Wound grade(after)**	**Size** **(before)**	**Size** **(after)**	**Duration**	**Additional procedure**	**Complication**
1	3	M	Ped TA	Dorsal	2	1	92	60	14	STSG	
2	7	M	Ped TA	Dorsal	3	1	23	9	17	STSG	scar contracture
3	7	M	Ped TA	Dorsal	2	1	60	42	16	STSG	scar contracture
4	10	M	Ped TA	Dorsal	3	1	36	16	13	STSG	
5	11	M	Sports injury	Lateral	4	1	27	20	23	STSG	
6	18	F	In car TA	Dorsal	2	1	70	50	19	STSG	
7	22	M	In car TA	Dorsal	3	1	94.5	87	17	STSG	
8	26	M	F/D	Medial	2	1	9	4	12	STSG	
9	27	M	In car TA	Dorsal	3	1	103	85	15	STSG	
10	44	M	Crushing	Dorsal	3	1	52	35	17	STSG	
11	47	F	Ped TA	Dorsal	2	1	151	91	27	STSG	
12	53	M	F/D	Medial	2	1	14	8	11	STSG	
13	54	F	Crushing	Medial	3	1	72.5	17	12	STSG	
14	63	F	Motorcycle TA	Dorsal	3	1	12	8	18	FTSG	
15	66	M	In car TA	Dorsal	3	3	45	39	21	free flap	
16	67	M	Ped TA	Dorsal	3	1	104	81	29	STSG	

**Mean**	**32** **(years-old)**				**2.69**	**1.13**	**56.4(cm**^2^)	**42.9(cm**^2^)	**18.4(days)**		

TA: traffic accident, Ped(pedestrian), F/D: fall down, STSG(split thickness skin graft), FTSG(full thickness skin graft)

### Technique

An NPWT device was applied after debriding necrotized tissues and cleansing contaminated wounds. When fractures were present, internal or external fixation was performed before application. The V.A.C.^®^system was used throughout. This consists of an evacuation tube, a collecting canister, a vacuum pump, and a multiporous polyurethane sponge, which directly contacts the wound. The sponge, which was designed to be 3–5 cm larger than wounds, was applied to defect sites and sealed with transparent cohesive film. The vacuum dressing was changed every 3–4 days and most procedures were performed at bedside. However, when necessary, debridement was performed in an operating room. A negative pressure vacuum pump was applied to wounds in continuous mode at a pressure of 100~125 mmHg. NPWT was stopped after confirming the formation of healthy granulation tissue. Skin grafting was performed when further coverage was required.

Wound types (acute or traumatic versus chronic) and location were noted, and durations, numbers, and frequencies of V.A.C. system applications were recorded. Before and after NPWT treatment, sizes of soft tissue defects were assessed using squared paper. Wounds were categorized into 5 groups based on degree of exposure and the presence of concomitant infection, which was graded from 0 to 4 (Table 2). Final coverage techniques, including primary closure, split thickness skin grafting, and pedicled local and vascularized free flap grafting were documented. Furthermore, any complications attributable to NPWT treatment were noted.

**Table 2:** Details of the open wound scoring system used

**Score (grade)**	**Status of wound**
0	Closed wound
1	Skin or soft tissue defect
2	Bone, tendon, implant exposure(any 1)
3	Bone, tendon, implant exposure(any combination of 2 or more)
4	Associated or Residual infection

## Results

**Figure 1:**
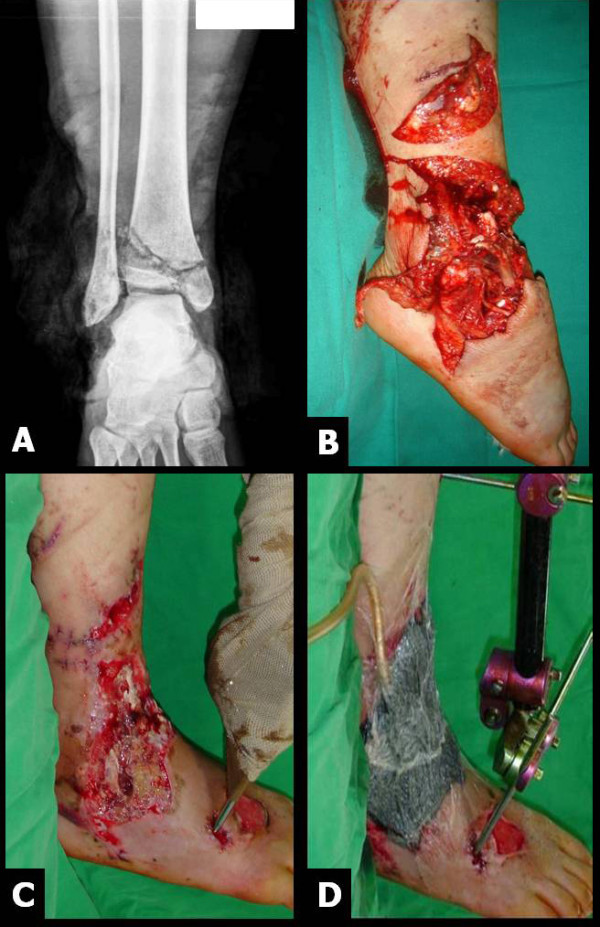
**A severe open fracture around the ankle in a 20 year-old woman (A & B)**. After the debridement of necrotized tissue (C), NPWT was applied (D).

**Figure 2:**
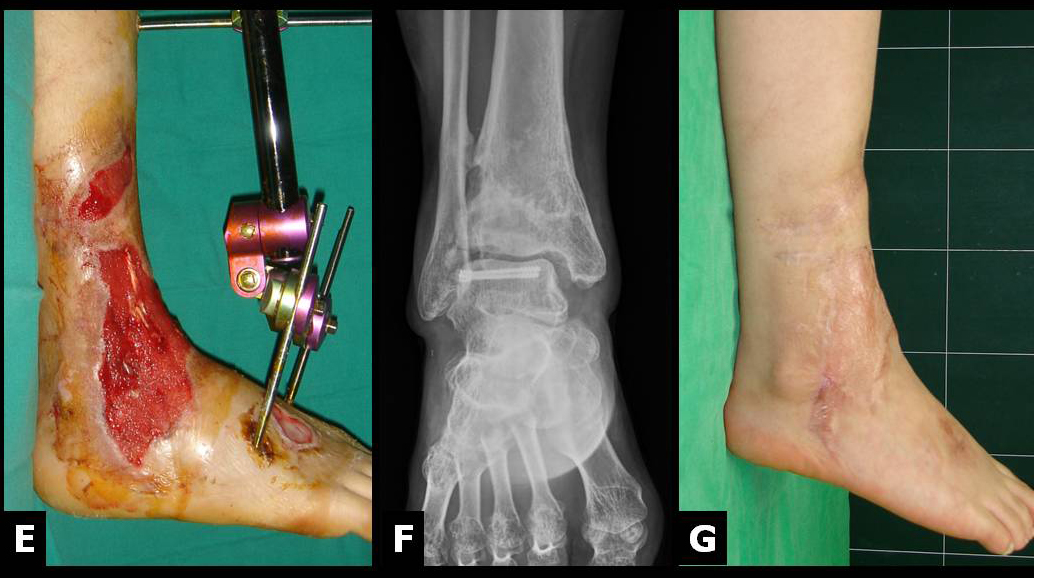
**At 22 days of NPWT, sufficient granulation covered tendons (E) to perform skin graft**. At 1 year postoperatively, the wound had healed well (F & G).

The mean duration of therapy was 18.4 days (range, 11–29 days), and dressings were changed 4.5 times on average. Mean wound size at treatment initiation was 56.4 cm^2 ^(9–151 cm^2^), and this reduced to 42.9 cm^2 ^(4–81 cm^2^) at treatment completion, an average wound area reduction of 24%. Fifteen of the 16 patients achieved an improved wound status, and in these exposed tendons or bone was covered with healthy granulation tissue (Figures 1, 2). After NPWT, skin grafting was performed to cover granulation tissue in 15 cases (a split-thickness skin graft in 14 cases and a full-thickness skin graft in 1 case). One patient experienced treatment failure, and required a free flap to cover exposed bone and tendon. The average wound grade was 2.69 at the start of treatment, and 1.13 at the end of treatment.

No complication occurred that could be directly attributed to NPWT, such as, a deep infection or bleeding. In terms of minor complications, four patients experienced itchiness of skin in the region of NPWT application. In addition, 2 patients experienced scar contractures in grafted areas, which were rescued using a releasing procedure.

## Discussion

Traumatic injuries around the foot and ankle are often associated with significant skin loss, which results in the exposure of tendons, bone, or hardware, and associated wound-management difficulties. These injuries are similar in many ways, to chronic ulcerative lesions of the foot associated with ischemic diseases, such as, diabetes mellitus. The rapid formation of granulation tissue and blood vessels are essential for the healing of these wounds. Traditionally, frequent wet dressing changes (3–4 times/day) are used to treat such cases, but this treatment is protracted and painful [3,7]. Furthermore, interstitial fluid from open wounds reduces local blood supply and disturbs wound healing due to its collagenase and metalloproteinase constituents [8,9]. From this viewpoint, NPWT is highly effective at clearing interstitial fluid, and in the majority of our patients, wounds were covered with healthy granulation tissue after 4.5 sponge changes, without additional flap surgery. DeFranzio^5 ^also reported that NPWT enhances rapid granulation formation in over 80% of patients as compared with a simple wet dressing. Furthermore, it has been well reported that NPWT provides a continuous physical stimulus that enhances the formation of new vessels and granulation tissues [10,11].

Soft tissue defects in the foot and ankle region usually require local or free flap surgery when a skin graft procedure is not applicable due to limited granulation tissue formation^1^. A split-thickness skin graft is not recommended for wounds with exposed bone or neurovascular structures, or for wounds involving the weight-bearing surface of the foot [12]. In a comparative study of traditional dressings and NPWT for lawnmower injuries of the lower leg [13], the need for free flap surgery was found to be decreased by 30%. A remarkable reduction in the requirement for secondary soft tissue operation is believed to be a big advantage of NPWT [14]. Dedmond [15] also reported that wounds of grade 3 with an accompanying open tibial fracture healed without the need for a secondary soft tissue operation, such as, a free flap. In the present study, the severities of open wounds were noticeably reduced after NPWT; only one patient needed a free flap to cover exposed bone and tendon.

The prevention of deep infection is essential during the treatment of soft tissue defects, and simple wet dressing may be inadequate in this context, because wounds are inevitably exposed to the atmosphere. On the other hand, NPWT not only seals open wounds but evacuates hematomas, exudates, and possible pathogens by the application of negative pressure [10,16,17]. Furthermore, it has been reported that NPWT is effective at treating deep infections [18]. In the present study, no case of infection during the treatment period occurred. Accordingly, we consider that NPWT probably also reduces soft tissue defect infection rates.

Some technical difficulties have been reported when NPWT was used to treat foot wounds [19], but we did not encounter these problems. In terms of complications, we did encounter 2 cases of skin graft scar contractures, which can reduce foot function. Successful scar release was achieved in these two cases. But, in certain cases, flap surgery may be considered to prevent scar contractures [20], instead of NPWT.

This study has several limitations that require consideration, namely, that the size of data is small, and there was no control group, which reduced objectivity. We suggest that a prospective randomized multicenter trial be undertaken to determine the merits of NPWT for the treatment of soft tissue defects of the ankle and foot. However, based on the results of previous studies on its use for the treatment of other injuries at other locations, it appears that NPWT plays a significant role in the formation of granulation tissue and in the prevention of infection [21].

Our results add to growing evidence that NPWT is a useful adjunctive treatment for open wounds around the foot and ankle. In the present study, it was found to facilitate the rapid formation of granulation tissue, to shorten healing time, and to reduce remarkably the need for additional soft tissue reconstructive surgery.

## Competing interests

The authors declare that they have no competing interests.

## Authors' contributions

CWO, HJL carried out concept design, patient recruitment and follow-up, data collection and analysis, and manuscript writing. JWK carried out literature search and data analysis. WKM carried out data collection, patient follow up, data analysis and manuscript writing. OJS, JKO, BCP, JCI conceived of the study, and participated in its design and coordination. All authors read and approved the final manuscript for publication.
